# Differentiation Between Glioblastoma Multiforme and Metastasis From the Lungs and Other Sites Using Combined Clinical/Routine MRI Radiomics

**DOI:** 10.3389/fcell.2021.710461

**Published:** 2021-08-26

**Authors:** Yuqi Han, Lingling Zhang, Shuzi Niu, Shuguang Chen, Bo Yang, Hongyan Chen, Fei Zheng, Yuying Zang, Hongbo Zhang, Yu Xin, Xuzhu Chen

**Affiliations:** ^1^School of Life Sciences and Technology, Xidian University, Xi’an, China; ^2^Key Laboratory of Molecular Imaging, Institute of Automation, Chinese Academy of Sciences, Beijing, China; ^3^Department of Radiology, Beijing Tiantan Hospital, Capital Medical University, Beijing, China; ^4^Institute of Software, Chinese Academy of Sciences, Beijing, China; ^5^School of Mathematical Sciences, Nankai University, Tianjin, China; ^6^Department of Computing, The Hong Kong Polytechnic University, Hong Kong, China; ^7^Department of Neurosurgery, Huizhou Third People’s Hospital, Guangzhou Medical University, Huizhou, China; ^8^Department of Neurosurgery, Beijing Tiantan Hospital, Capital Medical University, Beijing, China

**Keywords:** glioblastoma multiforme, metastasis, magnetic resonance imaging, machine learning, radiomics

## Abstract

**Background:**

Differentiation between cerebral glioblastoma multiforme (GBM) and solitary brain metastasis (MET) is important. The existing radiomic differentiation method ignores the clinical and routine magnetic resonance imaging (MRI) features.

**Purpose:**

To differentiate between GBM and MET and between METs from the lungs (MET-lung) and other sites (MET-other) through clinical and routine MRI, and radiomics analyses.

**Methods and Materials:**

A total of 350 patients were collected from two institutions, including 182 patients with GBM and 168 patients with MET, which were all proven by pathology. The ROI of the tumor was obtained on axial postcontrast MRI which was performed before operation. Seven radiomic feature selection methods and four classification algorithms constituted 28 classifiers in two classification strategies, with the best classifier serving as the final radiomics model. The clinical and combination models were constructed using the nomograms developed. The performance of the nomograms was evaluated in terms of calibration, discrimination, and clinical usefulness. Student’s *t*-test or the chi-square test was used to assess the differences in the clinical and radiological characteristics between the training and internal validation cohorts. Receiver operating characteristic curve analysis was performed to assess the performance of developed models with the area under the curve (AUC).

**Results:**

The classifier fisher_decision tree (fisher_DT) showed the best performance (AUC: 0.696, 95% CI:0.608-0.783) for distinguishing between GBM and MET in internal validation cohorts; the classifier reliefF_random forest (reliefF_RF) showed the best performance (AUC: 0.759, 95% CI: 0.613-0.904) for distinguishing between MET-lung and MET-other in internal validation cohorts. The combination models incorporating the radiomics signature and clinical-radiological characteristics were superior to the clinical-radiological models in the two classification strategies (AUC: 0.764 for differentiation between GBM in internal validation cohorts and MET and 0.759 or differentiation between MET-lung and MET-other in internal validation cohorts). The nomograms showed satisfactory performance and calibration and were considered clinically useful, as revealed in the decision curve analysis.

**Data Conclusion:**

The combination of radiomic and non-radiomic features is helpful for the differentiation among GBM, MET-lung, and MET-other.

## Introduction

Cerebral glioblastoma multiforme (GBM) and solitary brain metastasis (MET) are the most common brain tumors in adults (Ohgaki and Kleihues, 2005; Platta et al., 2010). Both GBM and MET show ring enhancement with peripheral edema on routine magnetic resonance imaging (MRI). Owing to the different treatment strategies available, a similar radiological appearance proposed a diagnostic dilemma for differentiation between the two lesions (Weller et al., 2014). Accurate differentiation between these two lesions is essential and has been one of the main focuses in radiological research for many years.

To date, studies aimed at the differentiation between GBM and MET have mainly shown two tendencies. The first tendency is to improve the performance of imaging modalities, and the second is to explore the differences among METs from different primary sites. For the first tendency, many imaging modalities have been proposed, including routine MRI and various advanced MRI modalities, such as magnetic resonance spectroscopy, diffusion-weighted imaging (DWI), diffusion tensor imaging, diffusion kurtosis imaging, perfusion-weighted imaging (PWI), arterial spin labeling, and amide proton transfer-weighted imaging (Chen et al., 2012; Tan et al., 2015; Salice et al., 2016; Durmo et al., 2018; Holly et al., 2018; Kamimura et al., 2019; Xi et al., 2019). For the second tendency, the relative cerebral blood volume showed no difference among METs from the lungs (MET-lung), breasts, gastrointestinal tract, and skin (Askaner et al., 2019). Another study showed that independent component analyses of dynamic susceptibility contrast PWI can show differences between breast MET and non-small-cell lung cancer (Chakhoyan et al., 2019). Moreover, breast METs were found to be less likely to be located in the posterior cerebral artery territory than MET-lung, kidneys, colon, and skin (Mampre et al., 2019).

Radiomics analysis has been proven to be useful in the diagnosis, prognosis assessment, and prediction of therapeutic responses in cancers by extracting exhaustive features from medical images (Aerts et al., 2014; Lambin et al., 2017). It has been used successfully in many studies of brain tumors, including those for tumor grading and genotype and overall survival assessment (Zacharaki et al., 2009; Grabowski et al., 2014; Li et al., 2018c; Chaddad et al., 2019). In particular, radiomics analysis was used to differentiate among METs from the breasts, lungs, and other sites in one study (Artzi et al., 2019). It was also used to differentiate METs among breast cancer, small-cell lung cancer, non-small-cell lung cancer, gastrointestinal cancer, and melanoma (Kniep et al., 2019). MET-lung, breasts, and skin also differed in texture features (Ortiz-Ramon et al., 2017; Ortiz-Ramón et al., 2018).

To date, the existing studies on the differentiation between GBM and MET have only focused on radiological data without consideration of clinical factors. Analyses of MET subtypes mainly considered the radiomic features without the routine MRI features. In addition, the subtypes of METs to be differentiated were not consistent in the existing studies. Of all METs, the top primary tumor is lung cancer (>50%) (Füreder et al., 2018; Rotta et al., 2018; Ascha et al., 2019). Therefore, we explored the differences between GBM and MET-lung and other sites (MET-other) with regard to the clinical and routine MRI and radiomic features in this study.

## Materials and Methods

This retrospective study was approved by the committees of two institutions; the need for obtaining informed consent from the patients was waived.

### Patients

Cerebral GBM was searched in the pathological database of our institution between January 2014 and December 2015. The inclusion and exclusion criteria are shown in Supplementary Material 1. A total of 152 patients with GBM were included from the first institution, and 30 patients with GBM were included from the second institution. All patients showed a supratentorial enhanced lesion in the cerebral parenchyma.

Solitary supratentorial MET was searched in the pathological database of the two institutions between January 2010 and December 2017. The inclusion and exclusion criteria are shown in Supplementary Material 1. Finally, a total of 76 patients with MET-lung and 62 patients with MET-other were included from the first institution, and 15 patients with MET-lung and 15 patients with MET-other were included from the second institution. The detailed primary cancers are shown in Supplementary Material 2.

### Image Acquisition and Analysis

All patients underwent MRI scanning within 2 weeks before cerebral operation. The tumor size was represented by the maximal diameter on the postcontrast axial image. Peritumoral edema was represented by the maximal diameter of the high signal around the tumor on the axial T2-weighted image (T2WI). The two parameters were manually measured using the Neurosoft PACS software^1^. The edema ratio was calculated by dividing the peritumoral edema by the tumor size. The location (left side/right side) was also reviewed by an experienced radiologist. The detailed scanning protocol and parameters are shown in Supplementary Material 3.

### Radiomics Analysis

#### Region of Interest (ROI) Segmentation

Using the ITK-SNAP software^2^ version 3.x, we opened the postcontrast axial sequence for each case and manually drew the outline of the enhanced lesion on each slice showing the tumor, which was saved as the segmented region of interest (ROI). The segmentation was performed by a radiologist with 14 years of experience and reviewed by another radiologist with 28 years of experience. Any discrepancy was resolved through discussion. The details of the drawing are illustrated in Supplementary Material 4.

#### Radiomic Feature Extraction

The radiomic features were extracted using PyRadiomics, which is an open-source python package for the extraction of radiomic features from medical images (Van Griethuysen et al., 2017). For each ROI, we extracted three types of radiomic features, including non-textural, textural, and wavelet features. The non-textural features included 13 shape features and 18 first-order features, and 74 textural features were calculated on the basis of 5 texture matrices: the gray level co-occurrence matrix (GLCM), gray level dependence matrix (GLDM), gray level run-length matrix (GLRLM), gray level size zone matrix (GLSZM), and neighborhood gray-tone difference matrix (NGTDM). The three-dimensional wavelet transformation decomposed the original image set into eight filtered images set in three directions. Finally, a total of 841 radiomic features were extracted, consisting of shape features in the original image, first-order features, and textural features in all images. A detailed description is provided in Supplementary Material 5.

#### Feature Reduction

First, we randomly selected 50 patients and translated (three pixels in the up, down, left, and right directions) and rotated (3° in clockwise and anticlockwise directions) their ROIs to evaluate the stability of the features through the intraclass correlation coefficients (threshold = 0.8). After the prescreening, all features were standardized using the z-scores derived from the training cohort. Thereafter, seven feature selection methods were used, including information theoretical-based feature selection: conditional mutual information maximization (CMIM), minimal-redundancy and maximal-relevance (MRMR), and double input symmetrical relevance (DISR); similarity-based feature selection: Fisher score and reliefF; and sparse learning-based feature selection: multi-cluster feature selection (MCFS) and robust feature selection (RFS), to recognize the most discriminating features. For each feature selection method, we ranked the features by their relevance score, and the best features were selected for the later classifiers.

#### Classifier Construction

Four algorithms were used to build the radiomics model: logistic regression (LR), support vector machine (SVM), decision tree (DT), and random forest (RF). These algorithms were implemented on the basis of the selected features and classification categories. The LR algorithm was used by tuning the regular term and penalty term. The SVM algorithm was used by tuning the penalty and gamma of the kernel function, where the kernel function is “rbf.” The DT algorithm was used by tuning two parameters: the maximum sample of the leaf and the maximum node. The RF algorithm was used by tuning the number of DTs and the maximum sample of the leaf. Fivefold cross validation was used for all 28 classifiers. The optimal classifier served as the final radiomics model. These algorithms were implemented using the Python version 3.6.5 “scikit-learn” package.

### Clinical-Radiological and Combination Models

The clinical characteristics (patient age and sex) and the routine radiological index (tumor size, edema ratio, and location) were used to construct the clinical-radiological model for differentiating between GBM and MET using an LR model (denoted as the clinical^*GBM*^ model). To distinguish MET-lung from MET-other, we used the same method to obtain the clinical-radiological model (denoted as the clinical^*MET*^ model). The clinical and routine radiological characteristics and radiomics signature were integrated to construct the combination models using the LR algorithm, and the optimal model was selected using AIC with a stepwise regression algorithm (denoted as the combination^*GBM*^ model and combination^*MET*^ model, respectively).

### Model Assessment

Receiver operating characteristic (ROC) curve analysis of each model was performed, and the areas under the curve (AUCs) were calculated in both the training and validation cohorts. The optimal cutoff value in the training cohort was applied to obtain accuracy, sensitivity, and specificity. The DeLong test was used to evaluate the statistical differences between the models. All assessments were performed in both the training and validation cohorts.

Nomogram analysis was applied to assess the potential clinical utility of the combination models. Calibration curves were drawn to evaluate the degree of deviation between the predictions and actual outcomes obtained using the Hosmer–Lemeshow test. Additionally, to evaluate the clinical utility of the nomograms, we performed a decision curve analysis by calculating the net benefits at different threshold probabilities (Rios Velazquez et al., 2017).

### Statistical Analysis

Patient age and sex, tumor size, and edema ratio were compared between the patients with GBM and MET and between those with MET-lung and MET-other using Student’s *t*-test or the chi-square test between the training, internal and external validation cohorts. *P-* values of <0.05 were considered to indicate a significant difference. The Spearman correlation coefficient was used to assess the relationship between the clinical and radiolocial characteristics and radiomic features. Statistical analysis was performed using IBM SPSS Statistics version 22.

## Results

### Clinical-Radiological Characteristics

The first institution included 152 patients with GBM and 138 patients with MET, which were randomly divided into a training cohort (*n* = 193) and an internal validation cohort (*n* = 97) with a ratio of 2:1. In addition, the patients with MET were also randomly divided into the training (*n* = 92) and validation cohorts (*n* = 46) at a ratio of 2:1. The second institute included 30 patients with GBM and 30 patients with MET, which were used as the external validation cohort.

The baseline characteristics are summarized in Table 1. There was no significant difference between the training and internal validation cohort for the two classification strategies. And, no significant difference between the training and external validation cohort for the two classification strategies.

**TABLE 1:** Cohort demographics.

**Cohort for differentiation of GBM and MET**					
**Characteristics**	**Training cohort (*n* = 193)**	**Internal validation cohort (*n* = 97)**	***p*-value**	**External validation cohort (*n* = 60)**	***p*-value**
Age [years, mean (SD)]	54.63 (11.63)	53.86 (13.39)	0.611	55.38 (11.45)	0.661
Sex [n (%)]			0.329		0.752
Male	117 (60.6)	53 (54.6)		35 (58.3)	
Female	76 (39.4)	44 (45.4)		25 (41.7)	
Diameter [mm, mean (SD)]	41.96 (14.88)	44.49 (16.10)	0.184	45.75 (14.80)	0.086
Location			0.902		—
Left	98 (50.8)	50 (51.5)		—	
Right	95 (49.2)	47 (48.5)		—	
Edema ratio [mean (SD)]	1.94 (0.79)	1.87 (0.82)	0.518	1.79 (0.66)	0.185

**Cohort for differentiation of MET-lung and MET-other**					
**Characteristics**	**Training cohort (*n* = 92)**	**Internal validation cohort (*n* = 46)**	***p*-value**	**External validation cohort (*n* = 30)**	***p*-value**

Age [years, mean (SD)]	57.93 (9.11)	57.50 (10.92)	0.805	55.27 (11.69)	0.198
Sex			0.714		
Male	53 (57.6)	28 (60.9)		16 (53.3)	0.682
Female	39 (42.4)	18 (39.1)		14 (46.7)	
Diameter [mm, mean (SD)]	36.10 (14.82)	37.28 (16.31)	0.669	42.03 (17.25)	0.070
Edema ratio [mean (SD)]	2.30 (0.83)	2.19 (1.08)	0.543	2.01 (0.81)	0.114
Location			0.717		—
Left	49 (53.3)	26 (56.5)		—	
Right	43 (46.7)	20 (43.5)		—	

*SD, standard deviation.*

### Radiomic Features

**FIGURE 1:**
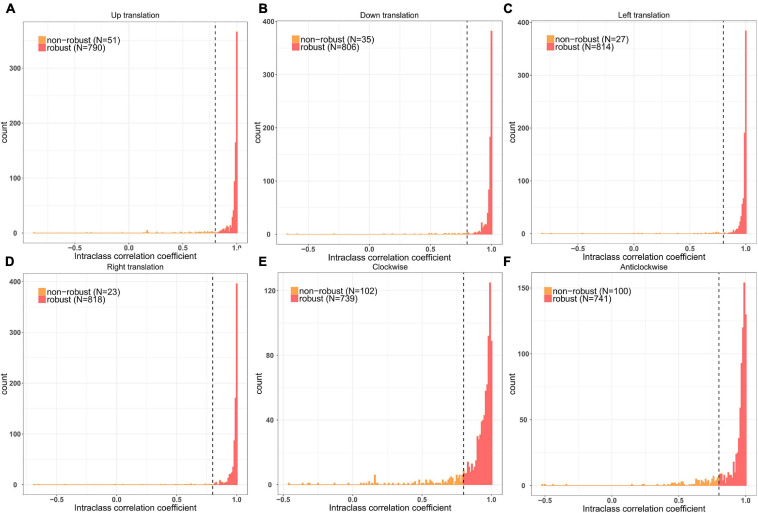
Remaining feature numbers after stability analyses for each disturbance. **(A,B)** The ROI was translated 3 pixels in the up and down directions; **(C,D)** The ROI was translated 3 pixels in the left and right directions; **(E,F)** The ROI was rotated 3° in clockwise and anticlockwise directions.

A total of 841 radiomic features were calculated for each patient. After prescreening using the intraclass correlation coefficients, 687 radiomic features with high stability were retained for subsequent analysis (Figure 1). Thereafter, the top 20 best features in each feature selection method were reserved to construct the radiomics models. Thus, seven feature subsets were formed for the two classification strategies. The detailed radiomic features are shown in Supplementary Material 6.

### Performance of the Radiomics Models

**FIGURE 2:**
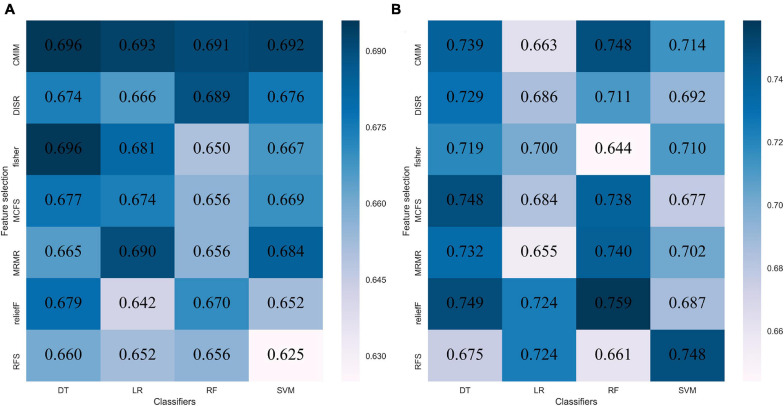
Performance of 28 classifiers in two classification strategies. **(A)** AUC of distinguishing GBM and MET in the internal validation cohort; **(B)** AUC of distinguishing MET-lung and MET-other in the external validation cohort. AUC, area under the curve; GBM, glioblastoma multiforme; MET, metastasis.

The performance of each of the 28 classifiers in the training and internal validation cohorts was reserved and is listed in Supplementary Material 7. For distinguishing between GBM and MET, the classifier fisher_decision tree (fisher_DT) showed the best performance in the internal validation cohort (AUC: 0.696, 95% CI: 0.608-0.783 Figure 2A and Supplementary Material 7). For differentiating between MET-lung and MET-other, the classifier reliefF_random forest (reliefF_RF) showed the best performance in the internal validation cohort (0.759,95% CI: 0.613-0.904, Figure 2B and Supplementary Material 7). The classifiers fisher_DT and reliefF_RF were selected as the optimal radiomics models in the two classification strategies, which were denoted as the radiomics^*GBM*^ model and radiomics^*MET*^ model, respectively. These two models are shown in Supplementary Material 8.

### Performance of the Clinical-Radiological and Combination Models

**FIGURE 3:**
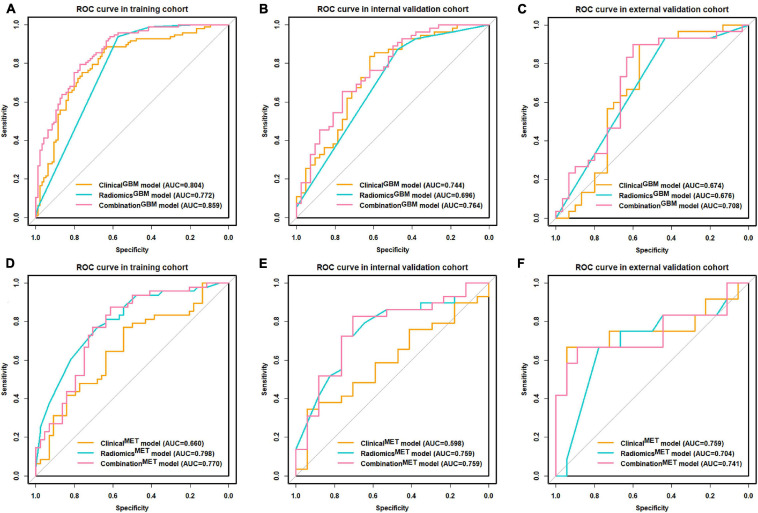
ROC curve for clinical, radiomics, and combination models. **(A–C)** ROC curves of distinguishing GBM and MET; **(D–F)** ROC curves of distinguishing MET-lung and MET-other. ROC, receiver operating characteristic; GBM, glioblastoma multiforme; MET, metastasis.

We summarized the performances of the clinical-radiological characteristics in the two classification strategies in Table 2. For distinguishing between GBM and MET, the clinical^*GBM*^ model exhibited satisfactory performance in all cohorts (training: AUC: 0.803, 95% CI: 0.740-0.867; internal validation: AUC: 0.744 95% CI: 0.643-0.846; external validation: AUC:0.674, 95% CI: 0.528-0.821, Figures 3A–C and Table 2). For distinguishing between MET-lung and MET-other, the AUC of the clinical^*MET*^ model was 0.598 (95% CI: 0.430-0.767) and 0.759 (95% CI: 0.548-0.971) in the internal and external validation cohort, respectively (Figures 3D–F and Table 2). For distinguishing between GBM and MET, the patient age, tumor diameter, edema ratio, and radiomics^*GBM*^ signature were considered as the input variables of the combination^*GBM*^ model after a stepwise search. For distinguishing between MET-lung and MET-other, the tumor diameter, edema ratio, and radiomics^*MET*^ signature were considered as the input variables of the combination^*MET*^ model. After the incorporation of the radiomics signatures, the performance of the combination models in the two classification strategies improved compared with that of the clinical models (Table 2). In particular, the performance of the combination^*MET*^ model was significantly better than that of the clinical^*MET*^ model (DeLong test: *P* = 0.019 in the internal validation cohort). The violin figures of all models in the training and validation cohorts are shown in Supplementary Material 9.

**TABLE 2:** Predictive performance of each model.

**Differentiation of GBM and MET**													
**Model**	**Traning cohort**				**Internal validation cohort**				**External validation cohort**				
	**AUC**	**ACC**	**SEN**	**SPE**	**AUC**	**ACC**	**SEN**	**SPE**	**AUC**	**ACC**		**SEN**	**SPE**
Clinical^*GBM*^ model	0.803 (0.740,0.867)	0.762	0.876	0.646	0.744 (0.643,0.846)	0.721	0.854	0.548	0.674 (0.528,0.821)	0.683		0.900	0.467
Radiomics^*GBM*^ model	0.772 (0.718,0.827)	0.757	0.938	0.573	0.696 (0.608,0.783)	0701	0.873	0.476	0.676 (0.572,0.779)	0.683		0.933	0.433
Combined^*GBM*^ model	0.859 (0.809,0.911)	0.783	0.794	0.771	0.764 (0.667,0.860)	0.691	0.655	0.738	0.708 (0.570,0.846)	0.617		0.567	0.667

**Differentiation of MET-lung and MET-other**													
**Model**	**Training cohort**				**Internal validation cohort**				**External validation cohort**				
	**AUC**	**ACC**	**SEN**	**SPE**	**AUC**	**ACC**	**SEN**	**SPE**	**AUC**	**ACC**	**SEN**		**SPE**

Clinical^*MET*^ model	0.660 (0.547,0.772)	0.663	0.771	0.546	0.598 (0.430,0.767)	0.457	0.621	0.177	0.759 (0.548,0.971)	0.700	0.750		0.667
Radiomics^*MET*^ model	0.798 (0.708,0.888)	0.728	0.771	0.682	0.759 (0.613, 0.904)	0.630	0.552	0.765	0.704 (0.492,0.901)	0.733	0.750		0.722
Combined^*MET*^ model	0.770 (0.672,0.869)	0.750	0.875	0.614	0.759 (0.609,0.908)	0.761	0.793	0.706	0.741 (0.527,0.954)	0.667	0.667		0.667

*ACC, accuracy; SEN, sensitivity; SPE, specificity.*

### Nomogram Implementation

**FIGURE 4:**
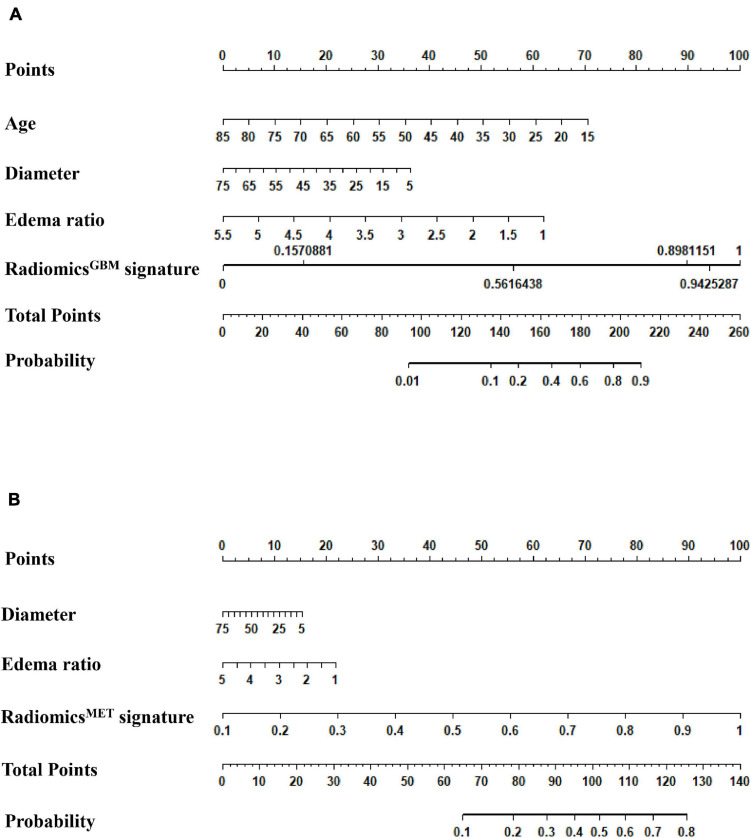
Model assessment. **(A)** Nomogram for distinguishing GBM and MET; **(B)** Nomogram for distinguishing MET-lung and MET-other. GBM, glioblastoma multiforme; MET, metastasis.

**FIGURE 5:**
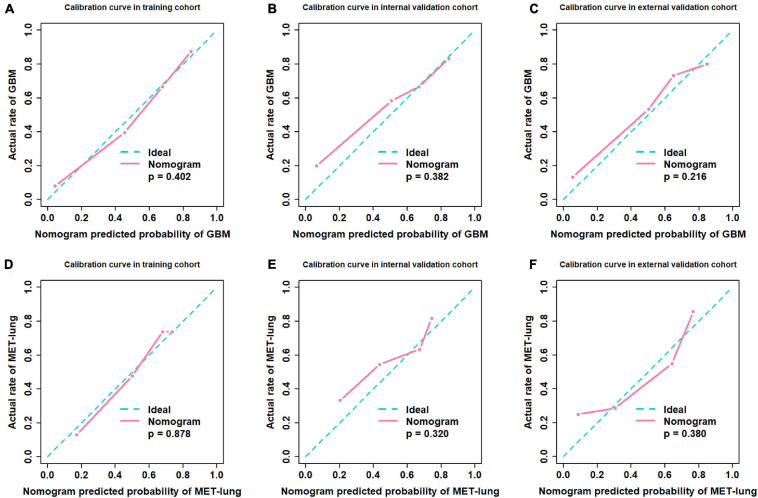
Calibration curves. **(A–C)** Calibration curves of distinguishing GBM and MET; **(D–F)** Calibration curves of distinguishing MET-lung and MET-other. GBM, glioblastoma multiforme; MET, metastasis.

**FIGURE 6:**
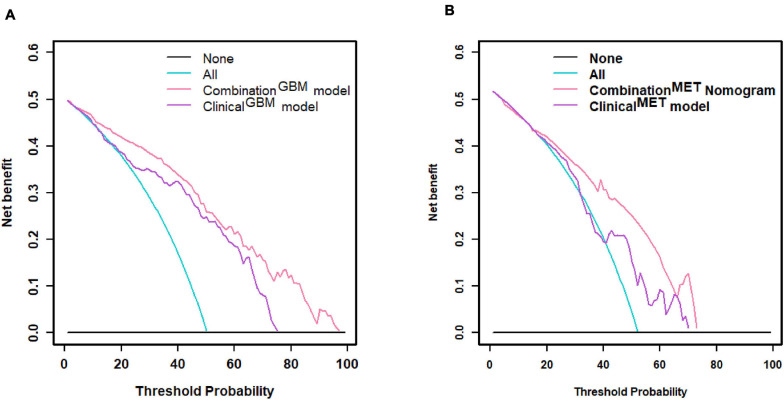
Decision curve. **(A)** Decision curve of distinguishing GBM and MET; **(B)** decision curve of distinguishing MET-lung and MET-other. GBM, glioblastoma multiforme; MET, metastasis.

We used the nomograms to show the graphical representation of the combination models. The nomograms for the two classification strategies are illustrated in Figure 4. The calibration curves demonstrated good agreement between the predictive and observational probabilities for the two classification strategies (*P* > 0.05 for all cohorts, Hosmer–Lemeshow test, Figure 5). The AUCs for the nomogram were 0.859 (95% CI: 0.809-0.911) in the training cohort, 0.764 (95% CI:0.667-0.860) in the internal validation cohort and 0.708 (95% CI: 0.570-0.846) in the external validation cohort for the differentiation between GBM and MET. The AUCs for the nomogram were 0.770 (95% CI: 0.672-0.869) in the training cohort, 0.759 (95% CI:0.609-0.908 in the internal validation cohort and 0.741 (95%CI:0.527-0.954) in the external validation cohort for the differentiation between MET-lung and MET-other. The decision curves showed that the combination GBM nomogram added more benefit than did the clinical^*GBM*^ nomogram when the threshold probability was >6% (Figure 6A); for the differentiation between MET-lung and MET-other, the combination^*MET*^ nomogram added more benefit than did the clinical^*MET*^ nomogram when the threshold probability was > 16% (Figure 6B). The correlation between the clinical-radiological characteristics and radiomic features was demonstrated in the heat map with the absolute value of the Spearman correlation coefficients (Supplementary Material 10).

## Discussion

We utilized radiomics analysis to distinguish between GBM and MET and between MET-lung and MET-other. For both classification strategies, we applied seven methods to select features and four algorithms to construct the radiomics model. Of all 28 classifiers for distinguishing between GBM and MET, the classifier fisher_DT exhibited the best classification performance, with an AUC of 0.696 in the internal validation cohort. For distinguishing between MET-lung and MET-other, the classifier reliefF_RF exhibited the best classification performance, with an AUC of 0.759 in the validation cohort. The combination models exhibited an improved predictive performance compared with the clinical models when the radiomics signatures were added to the models, especially for identifying the primary tumor of MET.

Radiomics analysis has been used for the differentiation between GBM and MET. To determine the best classification model for differentiation, 12 feature selection methods and 7 classification methods were used; the highest AUC obtained was 0.90 in the study by Qian et al. (2019). Artzi et al. (2019) used four machine-learning algorithms to differentiate between the GBM and MET subtypes, and the accuracies were 0.85, 0.89, 0.82, and 0.89 for identifying GBM and METs from breast, lung, and other cancers, respectively. All these studies have high clinical applicability but have only focused on the comparison of imaging features and radiomics models and did not consider the clinical factors. The complementarity of radiomic features and clinical-radiological factors should also be explored.

Considering the importance of patient age and sex in medical diagnosis, these variables were included in this study. Moreover, the tumor size, perilesional edema, and location are important radiological signs for diagnosis, which are readily obtained by routine radiological scans. The radiomics^*GBM*^ model and clinical^*GBM*^ model yielded a comparable predictive performance (*P* = 0.361 in the internal validation cohort, DeLong test). In addition, the predictive performance of the combination^*GBM*^ model improved compared with that of the clinical^*GBM*^ model when the radiomics^*GBM*^ signature and clinical-radiological factors were combined. However, the DeLong test showed no significant improvement (*P* = 0.064 in the internal validation cohort). This indicates that the radiomics signature can be used as a signal predictor to obtain satisfactory results. For differentiation between MET-lung and MET-other, and the performance of radiomics^*MET*^ model significantly better than that of the clinical^*MET*^ model (*P* = 0.019 in the internal validation cohort, DeLong test). The combination^*MET*^ model also showed a better predictive performance than the clinical^*MET*^ model, and the DeLong test showed significant improvements in the internal validation cohorts (*P* = 0.019), which suggested that the radiomics signature can increase the predictive power of clinical factors. Based on the results of the two classification strategies, we observed that radiomics analysis has a superior classification ability in differentiating tumor types, which is consistent with previous study findings (Artzi et al., 2019; Qian et al., 2019).

Our study also showed that the tumor size was related to the type of tumor, which was consistent with a previous study finding (Baris et al., 2016). Compared with the other characteristics, the tumor size had a higher correlation with the radiomic features used in the radiomics^*GBM*^ model (Supplementary Material 10), as observed in the Spearman correlation analysis. This may explain why the clinical^*GBM*^ model and radiomics^*GBM*^ model yielded a comparable predictive performance; however, the performance of the combination GBM model did not improve significantly, which emphasizes the importance of the tumor size in distinguishing between GBM and MET. With regard to the features used in the radiomics^*MET*^ model, most radiomic features showed low correlations with the clinical-radiological characteristics (Supplementary Material 10); thus, the performance of the combination^*MET*^ model improved significantly. This indicated that the radiomic features could complement the clinical factors, and the difference between MET-lung and MET-other could not be accurately recognized using simple tumor phenotypes.

We segmented tumors on post-contrast axial T1-weighted image (T1WI) not on other images, such as T2WI, et al. This is due to the different findings on different MRI images. For low-grade gliomas, they usually show no or partial enhancement, without or with minimal peritumoral edema. They are low signal intensity on post-contrast T1WI. Therefore, it is difficult to outline the border of the tumor on post-contrast T1WI images. On T2WI, however, low-grade gliomas are high signal intensity and prone to the identification of the tumor border. That is why many research studies segmented low-grade gliomas on T2WI (Li et al., 2018a,b; Liu et al., 2018, 2019; Qian et al., 2018). GBM, however, often shows a mass with vivid peritumoral edema. On post-contrast images, the mass usually demonstrates strong enhancement with non-enhanced peritumoral edema. Hence, the tumor mass is high signal while the peritumoral edema is low signal intensity on post-contrast T1WI, which is prone to outline the border of the mass. On T2WI, both the tumor itself and peritumoral edema are hyperintensity. It is difficult to distinguish the tumor from the perilesional edema. If the area of high signal intensity on T2WI is considered as the ROI for segmentation, the ROI would be larger than the tumor itself because the peritumoral edema is also recruited in the ROI. Cerebral metastasis often demonstrates as a mass with obvious edema. On T2WI and post-contrast T1WI, both the metastatic mass and the peritumoral edema show the same findings as that of GBM. Therefore, the radiomic analysis of cerebral metastasis is also based on post-contrast T1WI in some researches (Artzi et al., 2019; Karami et al., 2019a,b).

There are several limitations of this study. First, the radiomic imaging data used were only T1 enhanced sequences. Other sequences, including T2WI, DWI, and PWI, may contain additional functional and biological information; therefore, more imaging modalities should be taken into account for future research. Second, although the number of cases in our study was relatively large, the MET-other cases involved many origins, with each origin having a small case number. More detailed subgroups based on the primary origin of METs should be considered in future studies. Finally, this was a retrospective study. Although we used external validation to reduce the impact, the prospective multi-center study was still required.

## Conclusion

Our study suggests that radiomics analysis has a superior classification ability in the differentiation among GBM, MET-lung, and MET-other. The combination of radiomic and non-radiomic features is helpful for the differentiation of these three types of tumors.

## Data Availability Statement

The raw data supporting the conclusions of this article will be made available by the authors, without undue reservation.

## Ethics Statement

The studies involving human participants were reviewed and approved by Beijing Tiantan Hospital. Written informed consent for participation was not provided by the participants’ legal guardians/next of kin because: As a retrospective study, it was approved by our institute committee without the informed consent of the patients.

## Author Contributions

YH and LZ performed the study design, information collection, statistical analysis, and manuscript editing. HZ, YX, and XC guided and study design, reviewed images, and revised the manuscript. SN, SC, and BY provided the technical support. HC, FZ, and YZ collected the images and clinical information. All authors contributed to the article and approved the submitted version.

## Conflict of Interest

The authors declare that the research was conducted in the absence of any commercial or financial relationships that could be construed as a potential conflict of interest.

## Publisher’s Note

All claims expressed in this article are solely those of the authors and do not necessarily represent those of their affiliated organizations, or those of the publisher, the editors and the reviewers. Any product that may be evaluated in this article, or claim that may be made by its manufacturer, is not guaranteed or endorsed by the publisher.

## Footnotes

1
http://www.neusoft.com

2
http://www.itksnap.org/
