# Supplementary material for: Differentiation Between Glioblastoma Multiforme and Metastasis From the Lungs and Other Sites Using Combined Clinical/Routine MRI Radiomics
Source: Front Cell Dev Biol. 2021 Aug 26;9:710461. doi: 10.3389/fcell.2021.710461 (PMC8427511; doi:10.3389/fcell.2021.710461)
Supplement: Supplementary file 1 [file Data_Sheet_1.docx]

## S1: The inclusion and exclusion criteria of patient cohort

Fig. S1 Flow chart of the patient cohort with GBM

******

Fig. S2 Flow chart of the patient cohort with MET

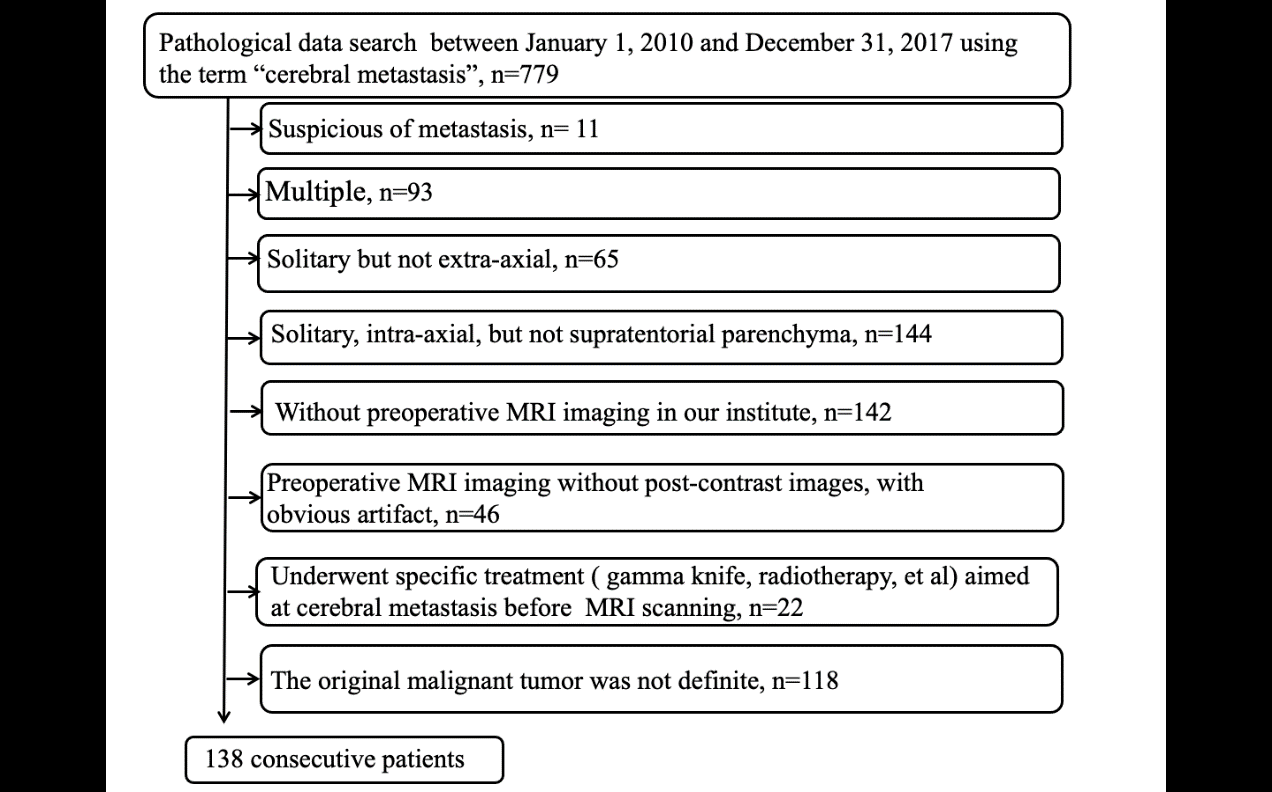


## S2: Summaries for subtypes of MET

A total of 138 patients with MET were recognized in this study, including 76 MET-lung and 62 MET-other. The primary malignant tumors are listed in following tables:

Table S1: Original cancers of solitary MET

| **Original cancers** | **Cases(n)** |
| --- | --- |
| Lung cancer | 76 |
| Breast cancer | 20 |
| Kidney cancer | 11 |
| Colorectal cancer | 10 |
| Metrocarcinoma | 4 |
| Esophagus cancer | 4 |
| Ovary cancer | 3 |
| Gastric cancer | 3 |
| Thyroid cancer | 1 |
| Bladder cancer | 1 |
| Oral squamous cell carcinoma | 1 |
| Calf melanoma | 1 |
| Adrenal carcinoma | 1 |
| Malignant thymoma | 1 |
| Pancreatic cancer | 1 |

## S3: The detailed MRI scanning protocol and parameters

For the 290 patients with cerebral operation (152 GBM and 138 MET), the scanning protocol included pre-and postcontrast scanning. The pre-contrast scanning included sagittal T1WI axial T1 and T2WI. Once the pre-contrast scanning was finished, the contrast media of dimeglumine gadopentetate (Beilu^®^, Beijing Beilu Pharmaceutical Co., LTD, China) was injected into the patient’s antecubital vein with a dose of 0.2ml/kg. After the injection, the scanning continued with axial, sagittal and coronal T1WI images obtained. The MR scanners are shown in the following table S2a,b.

Table S2a The MR scanner types in patients with GBM and MET in our institute

| **MR scanners** | **Tumors (n)** | |
| --- | --- | --- |
|  | **GBM** | **MET with operation** |
| **GE Discovery MR750** | 11 | 16 |
| **GE Medical System Genesis Signa** | 56 | 45 |
| **Siemens MAGNETOM Trio TimSystem** | 48 | 43 |
| **Siemens MAGNETOM Verio** | 37 | 34 |
| **Summary** | 152 | 138 |

Table S2b The MRI scanning parameters in patients with GBM and MET before operation in our institute

|  | **T2WI** | | | |
| --- | --- | --- | --- | --- |
|  | **TR(ms)** | **TE(ms)** | **Slice/Gap (mm)** | **Matrix** |
| **GE Discovery MR750** | 10221-10696 | 84 | 5.00/6.00 | 512×512 |
| **GE Medical System Genesis Signa** | 4900 | 116-117 | 5.00/6.00 | 512×512 |
| **Siemens MAGNETOM Trio TimSystem** | 4500 | 84 | 5.00/6.00 | 384×324 |
| **Siemens MAGNETOM Verio** | 6000 | 97 | 5.00/6.00 | 640×640 |
|  | Post-contrast axial T1WI | | | |
| **GE Discovery MR750** | 2804 | 19 | 5.00/6.00 | 512×512 |
| **GE Medical System Genesis Signa** | 2031 | 19 | 5.00/6.00 | 512×512 |
| **Siemens MAGNETOM Trio TimSystem** | 2000 | 9.8 | 5.00/6.00 | 512×432 |
| **Siemens MAGNETOM Verio** | 1900 | 9.4 | 5.00/6.00 | 512×496 |

## S4: The segmentation of region of interest (ROI).

**
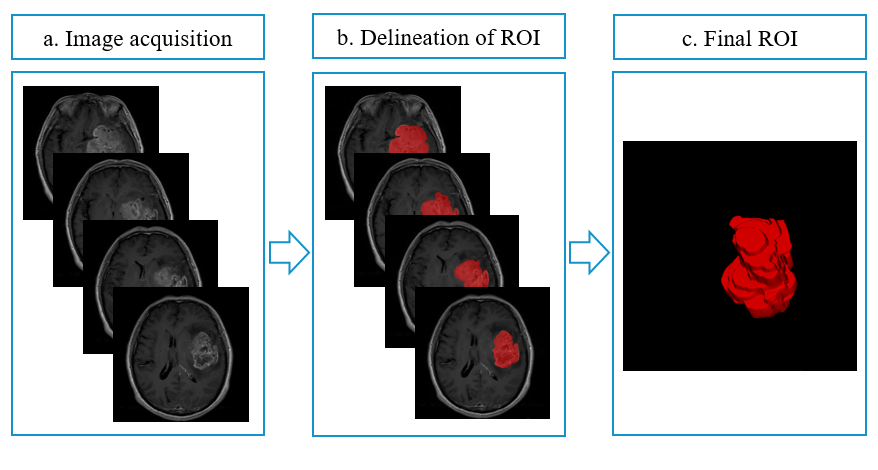
**

Fig. S3 a. All of the patients performed MRI examination on 3.0 T scanner; b. we delineated tumor regions along the boundaries on each 2-dimensional slicer with visible tumor through ITK-SNAP software; c. after the whole tumors were delineated, the union areas of the annotations was selected as the 3-dimensional ROI.

## S5: Definitions of radiomic features used in this study

Radiomic feature extraction of this study was based on Pyradiomics, including three types: non-textural, textural, and wavelet features. Non-textural features included the shape features (n=13) and first order statistical features (n=18). Textural features (n=74) were extracted based on five textural matrices: the Gray Level Co-occurrence Matrix (GLCM), the Gray Level Run-Length Matrix (GLRLM), the Gray Level Size Zone Matrix (GLSZM), the Neighborhood Gray-Tone Difference Matrix (NGTDM) and gray level dependence matrix (GLDM). The 3-dimensional wavelet transformation was applied to decompose the original image into 8 parts. The wavelet decompositions of original image were labeled as Wavelet.LLL, Wavelet.LLH, Wavelet.LHL, Wavelet.LHH, Wavelet.HLL, Wavelet.HLH, Wavelet.HLH, and Wavelet.HHH, where “L” and “H” represented low- and high-pass filters. For example, Wavelet.LLH represented the image filtered with low-pass in x- and y-directions and high-pass in z-directions, as follows:

$Wavelet.LLH(i,j,k)$= $\sum_{p=1}^{Nl} \sum_{q=1}^{Nl} \sum_{r=1}^{Nh} L(p)L(q)H(r)X(i+p, j+q,k+r)$,

where $Nl$ is the length of filter L, Nh is the length of filter H and X is the original image.

Types and names of the features are shown in Table S3. We finally extracted 841 radiomic features, including shape-based feature in original image, first order features and textural features in original and wavelet images (13+18*9+74*9 = 841). All the feature extraction was implemented using Python 3.6.5.

Table S3. Radiomics features used in this study.

| **Non-textural feature (n=31)** |
| --- |
| **Shape (n=13)** |
| Elongation |
| Flatness |
| Least Axis |
| Major Axis |
| Minor Axis |
| Maximum 2D Diameter Column |
| Maximum 2D Diameter Row |
| Maximum 2D Diameter Slice |
| Maximum 3D Diameter |
| Sphericity |
| Surface Area |
| Surface Volume Ratio |
| Volume |
| **First order features (n=18)** |
| 10th Percentile |
| 90th Percentile |
| Energy |
| Entropy |
| Interquartile Range |
| Kurtosis |
| Maximum |
| Mean |
| Mean Absolute Deviation |
| Median |
| Minimum |
| Range |
| Robust Mean Absolute Deviation |
| Root Mean Squared |
| Skewness |
| Total Energy |
| Uniformity |
| Variance |
| **Texture feature (n =74)** |
| **GLCM (n=23)** |
| Autocorrelation |
| Cluster Prominence |
| Cluster Shade |
| Cluster Tendency |
| Contrast |
| Correlation |
| Difference Average |
| Difference Entropy |
| Difference Variance |
| Inverse Difference (Id) |
| Inverse Difference Moment (Idm) |
| Inverse Difference Moment Normalized (Idmn) |
| Inverse Difference Normalized (Idn) |
| Informational Measure of Correlation 1 (Imc1) |
| Informational Measure of Correlation 2 (Imc2) |
| Inverse Variance |
| Joint Average |
| Joint Energy |
| Joint Entropy |
| Maximum Probability |
| Sum Average |
| Sum Entropy |
| Sum Squares |
| **GLDM (n=14)** |
| Dependence Entropy |
| Dependence Non-Uniformity |
| Dependence Non-Uniformity Normalized |
| Dependence Variance |
| Gray Level Non-Uniformity |
| Gray Level Variance |
| High Gray Level Emphasis |
| Large Dependence Emphasis |
| Large Dependence High Gray Level Emphasis |
| Large Dependence Low Gray Level Emphasis |
| Low Gray Level Emphasis |
| Small Dependence Emphasis |
| Small Dependence High Gray Level Emphasis |
| Small Dependence Low Gray Level Emphasis |
| **GLRLM (n=16)** |
| Gray Level Non-Uniformity |
| Gray Level Non-Uniformity Normalized |
| Gray Level Variance |
| High Gray Level Run Emphasis |
| Long Run Emphasis |
| Long Run High Gray Level Emphasis |
| Long Run Low Gray Level Emphasis |
| Low Gray Level Run Emphasis |
| Run Entropy |
| Run Length Non-Uniformity |
| Run Length Non-Uniformity Normalized |
| Run Percentage |
| Run Variance |
| Short Run Emphasis |
| Short Run High Gray Level Emphasis |
| Short Run Low Gray Level Emphasis |
| **GLSZM (n=16)** |
| Gray Level Non-Uniformity |
| Gray Level Non-Uniformity Normalized |
| Gray Level Variance |
| High Gray Level Zone Emphasis |
| Large Area Emphasis |
| Large Area High Gray Level Emphasis |
| Large Area Low Gray Level Emphasis |
| Low Gray Level Zone Emphasis |
| Size Zone Non-Uniformity |
| Size Zone Non-Uniformity Normalized |
| Small Area Emphasis |
| Small Area High Gray Level Emphasis |
| Small Area Low Gray Level Emphasis |
| Zone Entropy |
| Zone Percentage |
| Zone Variance |
| **NGTDM (n=5)** |
| Busyness |
| Coarseness |
| Complexity |
| Contrast |
| Strength |

## S6: Top 20 features for each method

Table S4a. Radiomic features of each method used in differentiation of GBM and MET

| **CMIM** | original_firstorder_Energy  original_firstorder_90Percentile  wavelet.HHH_glszm_GrayLevelNonUniformityNormalized  original_firstorder_InterquartileRange  wavelet.HHL_glszm_GrayLevelNonUniformityNormalized  wavelet.HHH_glszm_SizeZoneNonUniformity  wavelet.HHH_glszm_GrayLevelNonUniformity  original_firstorder_Median  wavelet.HHL_glszm_HighGrayLevelZoneEmphasis  wavelet.HHH_glszm_GrayLevelVariance  wavelet.HHL_glszm_GrayLevelVariance  wavelet.HHH_glszm_HighGrayLevelZoneEmphasis  wavelet.HHL_glszm_GrayLevelNonUniformity  original_firstorder_Range  wavelet.HHL_glszm_LowGrayLevelZoneEmphasis  original_firstorder_Maximum  wavelet.HHH_glszm_ZoneEntropy  wavelet.HHL_glszm_SizeZoneNonUniformity  wavelet.HHL_glszm_ZoneEntropy  wavelet.LLL_firstorder_Energy | **mRMR** | original_firstorder_Energy  wavelet.HHH_glszm_GrayLevelNonUniformityNormalized  wavelet.HHH_glszm_SizeZoneNonUniformity  original_firstorder_InterquartileRange  wavelet.HHH_glszm_GrayLevelVariance  wavelet.HHL_glszm_GrayLevelNonUniformityNormalized  wavelet.HHH_glszm_GrayLevelNonUniformity  wavelet.HHH_glszm_ZoneEntropy  wavelet.HHH_glszm_HighGrayLevelZoneEmphasis  wavelet.HHL_glszm_GrayLevelVariance  wavelet.HHL_glszm_SizeZoneNonUniformity  wavelet.HHL_glszm_GrayLevelNonUniformity  original_firstorder_Median  original_firstorder_Maximum  wavelet.HHL_glszm_HighGrayLevelZoneEmphasis  wavelet.HHL_glszm_SizeZoneNonUniformityNormalized  original_firstorder_90Percentile  wavelet.HHL_glszm_ZoneEntropy  original_firstorder_Range  wavelet.HHL_glszm_LowGrayLevelZoneEmphasis |
| --- | --- | --- | --- |
| **DISR** | original_firstorder_Energy  original_firstorder_90Percentile  original_firstorder_Entropy  original_firstorder_InterquartileRange  wavelet.HHH_glszm_GrayLevelNonUniformity  wavelet.HHH_glszm_SizeZoneNonUniformity  wavelet.HHL_glszm_SizeZoneNonUniformityNormalized  original_firstorder_Maximum  wavelet.HHH_firstorder_Maximum  wavelet.HHH_firstorder_Minimum  wavelet.HHH_firstorder_Range  wavelet.HHL_firstorder_Maximum  wavelet.HLH_firstorder_Maximum  wavelet.HLH_firstorder_Minimum  wavelet.HLH_firstorder_Range  wavelet.HLL_firstorder_Maximum  wavelet.HLL_firstorder_Minimum  wavelet.HLL_firstorder_Range  wavelet.LHH_firstorder_Maximum  wavelet.LHH_firstorder_Minimum | **ReliefF** | wavelet.HLH_glcm_Imc1  wavelet.HHL_glcm_InverseVariance  wavelet.LHH_glcm_Imc1  wavelet.LHL_glszm_SizeZoneNonUniformityNormalized  wavelet.LHL_glszm_SmallAreaEmphasis  original_shape_MajorAxis  wavelet.HHH_firstorder_InterquartileRange  wavelet.HLL_glszm_SmallAreaEmphasis  wavelet.HHL_firstorder_InterquartileRange  original_shape_Maximum2DDiameterColumn  wavelet.HLL_glszm_SizeZoneNonUniformityNormalized  wavelet.HHL_firstorder_RobustMeanAbsoluteDeviation  wavelet.HHH_firstorder_RobustMeanAbsoluteDeviation  original_shape_Maximum3DDiameter  wavelet.HLH_glszm_SmallAreaEmphasis  wavelet.HLH_glszm_SizeZoneNonUniformityNormalized  wavelet.LHH_glszm_SizeZoneNonUniformityNormalized  wavelet.HHL_firstorder_90Percentile  wavelet.LLH_gldm_DependenceEntropy  wavelet.HHL_firstorder_MeanAbsoluteDeviation |
| **Fisher** | original_shape_MajorAxis  original_shape_Maximum3DDiameter  original_shape_Maximum2DDiameterSlice  original_shape_Maximum2DDiameterColumn  original_shape_MinorAxis  original_shape_Maximum2DDiameterRow  original_shape_SurfaceArea  original_shape_LeastAxis  wavelet.LHL_glszm_SizeZoneNonUniformityNormalized  wavelet.HLH_glszm_SizeZoneNonUniformityNormalized  wavelet.LHH_glszm_SizeZoneNonUniformityNormalized  wavelet.HLH_glszm_SmallAreaEmphasis  wavelet.HLL_glszm_SizeZoneNonUniformityNormalized  wavelet.LHL_glszm_SmallAreaEmphasis  wavelet.HLL_glszm_SmallAreaEmphasis  wavelet.LHH_glszm_SmallAreaEmphasis  original_shape_Sphericity  original_shape_Volume  wavelet.LLH_glszm_ZoneEntropy  wavelet.HHL_firstorder_InterquartileRange | **RFS** | original_shape_MajorAxis  wavelet.HLL_glcm_InverseVariance  wavelet.LHL_glszm_SmallAreaEmphasis  wavelet.HHL_gldm_DependenceVariance  wavelet.LHH_glszm_SmallAreaEmphasis  wavelet.HHH_gldm_LargeDependenceEmphasis  wavelet.HLH_glszm_SmallAreaEmphasis  original_shape_MinorAxis  original_glcm_Imc2  wavelet.LLH_glcm_DifferenceAverage  original_glcm_Idmn  wavelet.HLL_glszm_ZoneEntropy  original_shape_Maximum2DDiameterSlice  original_shape_SurfaceArea  wavelet.LLH_firstorder_TotalEnergy  wavelet.HHH_ngtdm_Busyness  wavelet.HHH_glrlm_LongRunLowGrayLevelEmphasis  wavelet.LHH_glszm_ZoneEntropy  original_shape_Maximum3DDiameter  wavelet.LHH_glcm_Imc1 |
| **MCFS** | wavelet.LHH_glszm_ZoneVariance  original_glcm_JointEntropy  original_glcm_Autocorrelation  wavelet.LHH_glcm_DifferenceEntropy  wavelet.HHL_gldm_LowGrayLevelEmphasis  wavelet.HHH_ngtdm_Coarseness  original_glcm_DifferenceEntropy  wavelet.LHL_gldm_DependenceNonUniformity  wavelet.HLL_glcm_DifferenceAverage  wavelet.HLH_glrlm_LongRunHighGrayLevelEmphasis  wavelet.LHH_glcm_JointAverage  wavelet.LHL_glrlm_RunVariance  wavelet.LLL_firstorder_Maximum  wavelet.LLL_gldm_DependenceEntropy  wavelet.LLL_glcm_SumEntropy  wavelet.LLL_glcm_SumAverage  wavelet.HHH_firstorder_Energy  wavelet.HHH_glcm_Idm  wavelet.HHL_firstorder_InterquartileRange  wavelet.LHH_glrlm_RunPercentage |  |  |

Table S4b. Radiomic features of each method used in differentiation of MET-lung and MET-other

| **CMIM** | original_firstorder_Energy  original_firstorder_Median  wavelet.HHH_glszm_GrayLevelNonUniformity  original_firstorder_InterquartileRange  wavelet.HHH_glszm_ZoneEntropy  wavelet.HHH_glszm_GrayLevelNonUniformityNormalized  wavelet.HHH_glszm_SizeZoneNonUniformity  wavelet.HHL_glszm_GrayLevelVariance  original_firstorder_Maximum  wavelet.HHH_glszm_GrayLevelVariance  wavelet.HHL_glszm_GrayLevelNonUniformityNormalized  wavelet.HHH_glszm_HighGrayLevelZoneEmphasis  wavelet.HHL_glszm_ZoneEntropy  original_firstorder_Range  wavelet.HHL_glszm_SizeZoneNonUniformity  wavelet.HHL_glszm_HighGrayLevelZoneEmphasis  wavelet.HHL_glszm_GrayLevelNonUniformity  wavelet.HHL_glszm_LowGrayLevelZoneEmphasis  wavelet.HHL_glszm_SizeZoneNonUniformityNormalized  original_shape_Maximum2DDiameterRow | **mRMR** | original_firstorder_Energy  wavelet.HHH_glszm_SizeZoneNonUniformity  wavelet.HHH_glszm_GrayLevelNonUniformityNormalized  wavelet.HHL_glszm_GrayLevelNonUniformityNormalized  wavelet.HHH_glszm_GrayLevelNonUniformity  original_firstorder_InterquartileRange  wavelet.HHH_glszm_ZoneEntropy  wavelet.HHH_glszm_GrayLevelVariance  wavelet.HHL_glszm_GrayLevelNonUniformity  wavelet.HHH_glszm_HighGrayLevelZoneEmphasis  original_firstorder_90Percentile  original_firstorder_Median  wavelet.HHL_glszm_GrayLevelVariance  wavelet.HHL_glszm_SizeZoneNonUniformity  original_shape_Maximum2DDiameterRow  wavelet.HHL_glszm_HighGrayLevelZoneEmphasis  wavelet.HHL_glszm_SizeZoneNonUniformityNormalized  original_shape_Maximum2DDiameterSlice  wavelet.HHL_glszm_LowGrayLevelZoneEmphasis  original_firstorder_Maximum |
| --- | --- | --- | --- |
| **DISR** | original_firstorder_Energy  original_firstorder_90Percentile  original_firstorder_Entropy  original_firstorder_InterquartileRange  wavelet.HHL_glszm_GrayLevelNonUniformityNormalized  original_firstorder_Maximum  original_firstorder_Range  original_firstorder_Mean  original_firstorder_MeanAbsoluteDeviation  original_firstorder_Median  original_firstorder_RobustMeanAbsoluteDeviation  original_firstorder_RootMeanSquared  original_firstorder_TotalEnergy  original_firstorder_Uniformity  original_firstorder_Variance  original_glcm_Autocorrelation  original_glcm_Contrast  original_glcm_DifferenceAverage  original_glcm_DifferenceEntropy  original_glcm_DifferenceVariance | **ReliefF** | wavelet.HLH_gldm_LargeDependenceLowGrayLevelEmphasis  wavelet.HLH_glszm_LowGrayLevelZoneEmphasis  wavelet.LHH_glrlm_ShortRunLowGrayLevelEmphasis  original_glszm_LargeAreaHighGrayLevelEmphasis  wavelet.LLL_firstorder_Kurtosis  wavelet.LHH_glszm_SmallAreaLowGrayLevelEmphasis  original_glszm_SizeZoneNonUniformityNormalized  wavelet.LLH_glcm_SumEntropy  wavelet.LLL_gldm_LargeDependenceHighGrayLevelEmphasis  wavelet.HLH_gldm_LowGrayLevelEmphasis  wavelet.HLH_glrlm_LowGrayLevelRunEmphasis  wavelet.LLH_glszm_SizeZoneNonUniformityNormalized  wavelet.LLL_glcm_DifferenceEntropy  wavelet.LHH_gldm_LowGrayLevelEmphasis  wavelet.HLL_glszm_LowGrayLevelZoneEmphasis  wavelet.LLH_glrlm_RunEntropy  wavelet.LHH_glrlm_LowGrayLevelRunEmphasis  wavelet.LLH_firstorder_Entropy  wavelet.LLH_glszm_SmallAreaEmphasis  wavelet.LLH_ngtdm_Strength |
| **Fisher** | wavelet.LLH_gldm_DependenceEntropy  wavelet.LLH_glszm_ZoneEntropy  wavelet.LLH_glrlm_RunEntropy  wavelet.LLH_firstorder_10Percentile  wavelet.LLH_firstorder_Maximum  original_glrlm_RunEntropy  wavelet.LLH_firstorder_Energy  wavelet.LLL_glrlm_RunEntropy  original_gldm_DependenceEntropy  wavelet.LLL_glcm_Autocorrelation  wavelet.LLL_glszm_ZoneEntropy  wavelet.LLL_glcm_SumAverage  wavelet.LLL_glcm_JointAverage  wavelet.LLH_firstorder_Entropy  wavelet.LLL_glcm_DifferenceAverage  wavelet.LLL_gldm_DependenceEntropy  wavelet.LLL_glrlm_LongRunHighGrayLevelEmphasis  wavelet.LLH_firstorder_RootMeanSquared  wavelet.LLL_gldm_HighGrayLevelEmphasis  wavelet.LLL_glrlm_HighGrayLevelRunEmphasis | **RFS** | wavelet.HLL_glcm_InverseVariance  wavelet.LLH_gldm_DependenceEntropy  wavelet.LLL_gldm_LargeDependenceHighGrayLevelEmphasis  wavelet.LLH_glszm_ZoneEntropy  wavelet.LHH_glcm_Imc1  wavelet.LLH_firstorder_Maximum  wavelet.LLL_glrlm_LongRunHighGrayLevelEmphasis  original_gldm_LargeDependenceHighGrayLevelEmphasis  wavelet.LLL_glcm_Autocorrelation  wavelet.LLL_glszm_HighGrayLevelZoneEmphasis  original_glrlm_LongRunHighGrayLevelEmphasis  wavelet.LLL_glcm_JointAverage  wavelet.LLL_glcm_SumAverage  wavelet.LLH_firstorder_Energy  wavelet.LLL_glrlm_HighGrayLevelRunEmphasis  wavelet.LLL_gldm_HighGrayLevelEmphasis  wavelet.HLH_glcm_Imc1  wavelet.LLL_glrlm_ShortRunHighGrayLevelEmphasis  wavelet.HHH_gldm_LargeDependenceEmphasis  wavelet.LLL_glszm_GrayLevelVariance |
| **MCFS** | wavelet.HHL_glcm_SumEntropy  wavelet.LLH_glrlm_RunPercentage  wavelet.HHH_firstorder_InterquartileRange  wavelet.LHH_glrlm_LongRunLowGrayLevelEmphasis  wavelet.LHH_glrlm_LongRunEmphasis  wavelet.LHH_gldm_LowGrayLevelEmphasis  wavelet.LHH_gldm_LargeDependenceLowGrayLevelEmphasis  wavelet.HHH_glcm_SumAverage  wavelet.HLL_glszm_ZonePercentage  wavelet.HLL_glszm_LargeAreaEmphasis  wavelet.HLL_glrlm_RunVariance  wavelet.HHH_gldm_LargeDependenceHighGrayLevelEmphasis  wavelet.HLL_gldm_LargeDependenceLowGrayLevelEmphasis  wavelet.HLL_gldm_DependenceVariance  wavelet.HLL_glcm_SumSquares  original_glcm_JointAverage  wavelet.HLL_glcm_SumAverage  wavelet.HLL_glcm_JointEnergy  wavelet.LLL_glcm_Imc1  wavelet.HLL_glcm_JointAverage |  |  |

## S7: Performance of 28 classifiers both in training and validation cohorts

Table S5a. Results for differentiation of GBM and MET

| **Feature selection** | **Classifiers** | **Training cohort** | | | | **Validation cohort** | | | |
| --- | --- | --- | --- | --- | --- | --- | --- | --- | --- |
|  |  | **AUC** | **ACC** | **SEN** | **SPE** | **AUC** | **ACC** | **SEN** | **SPE** |
| **CMIM** | **DT** | 0.935 | 0.927 | 1.000 | 0.854 | 0.696 | 0.701 | 0.836 | 0.524 |
|  | **LR** | 0.705 | 0.668 | 0.701 | 0.635 | 0.693 | 0.660 | 0.727 | 0.571 |
|  | **RF** | 0.989 | 0.953 | 0.948 | 0.958 | 0.691 | 0.639 | 0.655 | 0.619 |
|  | **SVM** | 0.782 | 0.777 | 0.794 | 0.760 | 0.692 | 0.619 | 0.582 | 0.667 |
| **DISR** | **DT** | 0.801 | 0.772 | 0.918 | 0.625 | 0.674 | 0.660 | 0.818 | 0.452 |
|  | **LR** | 0.681 | 0.674 | 0.753 | 0.594 | 0.666 | 0.619 | 0.745 | 0.452 |
|  | **RF** | 0.920 | 0.881 | 0.959 | 0.802 | 0.689 | 0.680 | 0.727 | 0.619 |
|  | **SVM** | 0.779 | 0.741 | 0.701 | 0.781 | 0.676 | 0.588 | 0.582 | 0.595 |
| **fisher** | **DT** | 0.772 | 0.756 | 0.938 | 0.573 | 0.696 | 0.701 | 0.873 | 0.476 |
|  | **LR** | 0.848 | 0.782 | 0.660 | 0.906 | 0.681 | 0.608 | 0.527 | 0.714 |
|  | **RF** | 0.883 | 0.803 | 0.753 | 0.854 | 0.650 | 0.526 | 0.473 | 0.595 |
|  | **SVM** | 0.891 | 0.860 | 0.784 | 0.938 | 0.667 | 0.567 | 0.382 | 0.810 |
| **MCFS** | **DT** | 0.790 | 0.767 | 0.918 | 0.615 | 0.677 | 0.691 | 0.855 | 0.476 |
|  | **LR** | 0.762 | 0.736 | 0.722 | 0.750 | 0.674 | 0.639 | 0.673 | 0.595 |
|  | **RF** | 0.834 | 0.762 | 0.711 | 0.813 | 0.656 | 0.629 | 0.600 | 0.667 |
|  | **SVM** | 0.785 | 0.756 | 0.732 | 0.781 | 0.669 | 0.629 | 0.636 | 0.619 |
| **mRMR** | **DT** | 0.794 | 0.736 | 0.732 | 0.740 | 0.665 | 0.639 | 0.691 | 0.571 |
|  | **LR** | 0.743 | 0.710 | 0.722 | 0.698 | 0.690 | 0.629 | 0.655 | 0.595 |
|  | **RF** | 0.734 | 0.705 | 0.701 | 0.708 | 0.656 | 0.649 | 0.691 | 0.595 |
|  | **SVM** | 0.639 | 0.642 | 0.897 | 0.385 | 0.684 | 0.649 | 0.836 | 0.405 |
| **reliefF** | **DT** | 0.796 | 0.767 | 0.866 | 0.667 | 0.679 | 0.660 | 0.745 | 0.548 |
|  | **LR** | 0.791 | 0.767 | 0.845 | 0.688 | 0.642 | 0.639 | 0.673 | 0.595 |
|  | **RF** | 0.848 | 0.777 | 0.784 | 0.771 | 0.670 | 0.649 | 0.709 | 0.571 |
|  | **SVM** | 0.702 | 0.689 | 0.546 | 0.833 | 0.652 | 0.629 | 0.455 | 0.857 |
| **RFS** | **DT** | 0.748 | 0.736 | 0.887 | 0.583 | 0.660 | 0.670 | 0.818 | 0.476 |
|  | **LR** | 0.746 | 0.736 | 0.907 | 0.563 | 0.652 | 0.680 | 0.818 | 0.500 |
|  | **RF** | 0.745 | 0.736 | 0.907 | 0.563 | 0.656 | 0.680 | 0.818 | 0.500 |
|  | **SVM** | 0.998 | 0.995 | 1.000 | 0.990 | 0.625 | 0.546 | 0.473 | 0.643 |

Table S5b. Results for differentiation of MET-lung and MET-other

| **Feature selection** | **Classifiers** | **Training cohort** | | | | **Validation cohort** | | | |
| --- | --- | --- | --- | --- | --- | --- | --- | --- | --- |
|  |  | **AUC** | **ACC** | **SEN** | **SPE** | **AUC** | **ACC** | **SEN** | **SPE** |
| **CMIM** | **DT** | 0.884 | 0.859 | 0.979 | 0.727 | 0.739 | 0.717 | 0.828 | 0.529 |
|  | **LR** | 0.637 | 0.641 | 0.854 | 0.409 | 0.663 | 0.696 | 0.828 | 0.471 |
|  | **RF** | 0.757 | 0.707 | 0.708 | 0.705 | 0.748 | 0.630 | 0.552 | 0.765 |
|  | **SVM** | 0.745 | 0.728 | 0.750 | 0.705 | 0.714 | 0.565 | 0.448 | 0.765 |
| **DISR** | **DT** | 0.949 | 0.891 | 1.000 | 0.773 | 0.729 | 0.652 | 0.690 | 0.588 |
|  | **LR** | 0.761 | 0.717 | 0.958 | 0.455 | 0.686 | 0.609 | 0.793 | 0.294 |
|  | **RF** | 0.821 | 0.772 | 0.729 | 0.818 | 0.711 | 0.543 | 0.379 | 0.824 |
|  | **SVM** | 0.345 | 0.533 | 1.000 | 0.023 | 0.692 | 0.630 | 0.966 | 0.059 |
| **fisher** | **DT** | 0.984 | 0.946 | 0.938 | 0.955 | 0.719 | 0.630 | 0.586 | 0.706 |
|  | **LR** | 0.613 | 0.609 | 0.646 | 0.568 | 0.700 | 0.630 | 0.621 | 0.647 |
|  | **RF** | 0.734 | 0.674 | 0.854 | 0.477 | 0.644 | 0.674 | 0.724 | 0.588 |
|  | **SVM** | 0.368 | 0.533 | 0.938 | 0.091 | 0.710 | 0.696 | 0.966 | 0.235 |
| **MCFS** | **DT** | 0.808 | 0.783 | 1.000 | 0.545 | 0.748 | 0.674 | 0.862 | 0.353 |
|  | **LR** | 0.639 | 0.630 | 0.875 | 0.364 | 0.684 | 0.543 | 0.793 | 0.118 |
|  | **RF** | 0.964 | 0.913 | 0.979 | 0.841 | 0.738 | 0.717 | 0.759 | 0.647 |
|  | **SVM** | 0.685 | 0.674 | 0.958 | 0.364 | 0.677 | 0.543 | 0.793 | 0.118 |
| **mRMR** | **DT** | 0.898 | 0.848 | 0.792 | 0.909 | 0.732 | 0.652 | 0.621 | 0.706 |
|  | **LR** | 0.590 | 0.598 | 0.708 | 0.477 | 0.655 | 0.587 | 0.621 | 0.529 |
|  | **RF** | 0.939 | 0.870 | 0.833 | 0.909 | 0.740 | 0.609 | 0.552 | 0.706 |
|  | **SVM** | 0.778 | 0.761 | 0.708 | 0.818 | 0.702 | 0.565 | 0.414 | 0.824 |
| **reliefF** | **DT** | 0.770 | 0.728 | 1.000 | 0.432 | 0.749 | 0.739 | 0.897 | 0.471 |
|  | **LR** | 0.656 | 0.641 | 0.500 | 0.795 | 0.724 | 0.674 | 0.586 | 0.824 |
|  | **RF** | 0.798 | 0.728 | 0.771 | 0.682 | 0.759 | 0.630 | 0.552 | 0.765 |
|  | **SVM** | 0.637 | 0.652 | 0.542 | 0.773 | 0.687 | 0.652 | 0.586 | 0.765 |
| **RFS** | **DT** | 1.000 | 0.989 | 0.979 | 1.000 | 0.675 | 0.652 | 0.586 | 0.765 |
|  | **LR** | 0.643 | 0.652 | 0.792 | 0.500 | 0.724 | 0.674 | 0.724 | 0.588 |
|  | **RF** | 0.736 | 0.696 | 0.938 | 0.432 | 0.661 | 0.674 | 0.897 | 0.294 |
|  | **SVM** | 0.681 | 0.674 | 0.792 | 0.545 | 0.748 | 0.717 | 0.793 | 0.588 |

## S8: Visualization for fisher_DT model and reliefF_RF model

**
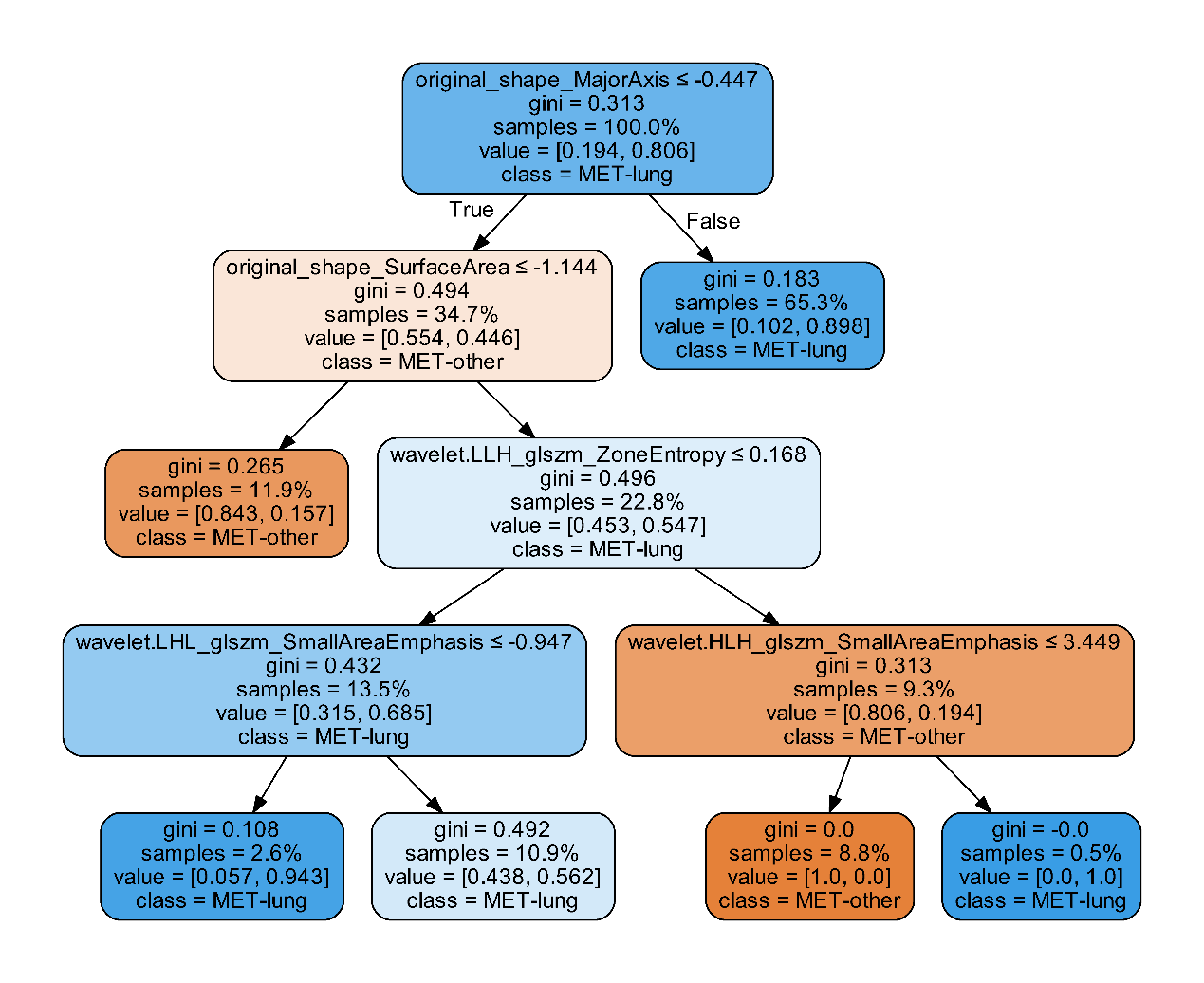
**

Fig. S4 fisher_DT model for differentiation of GBM and MET


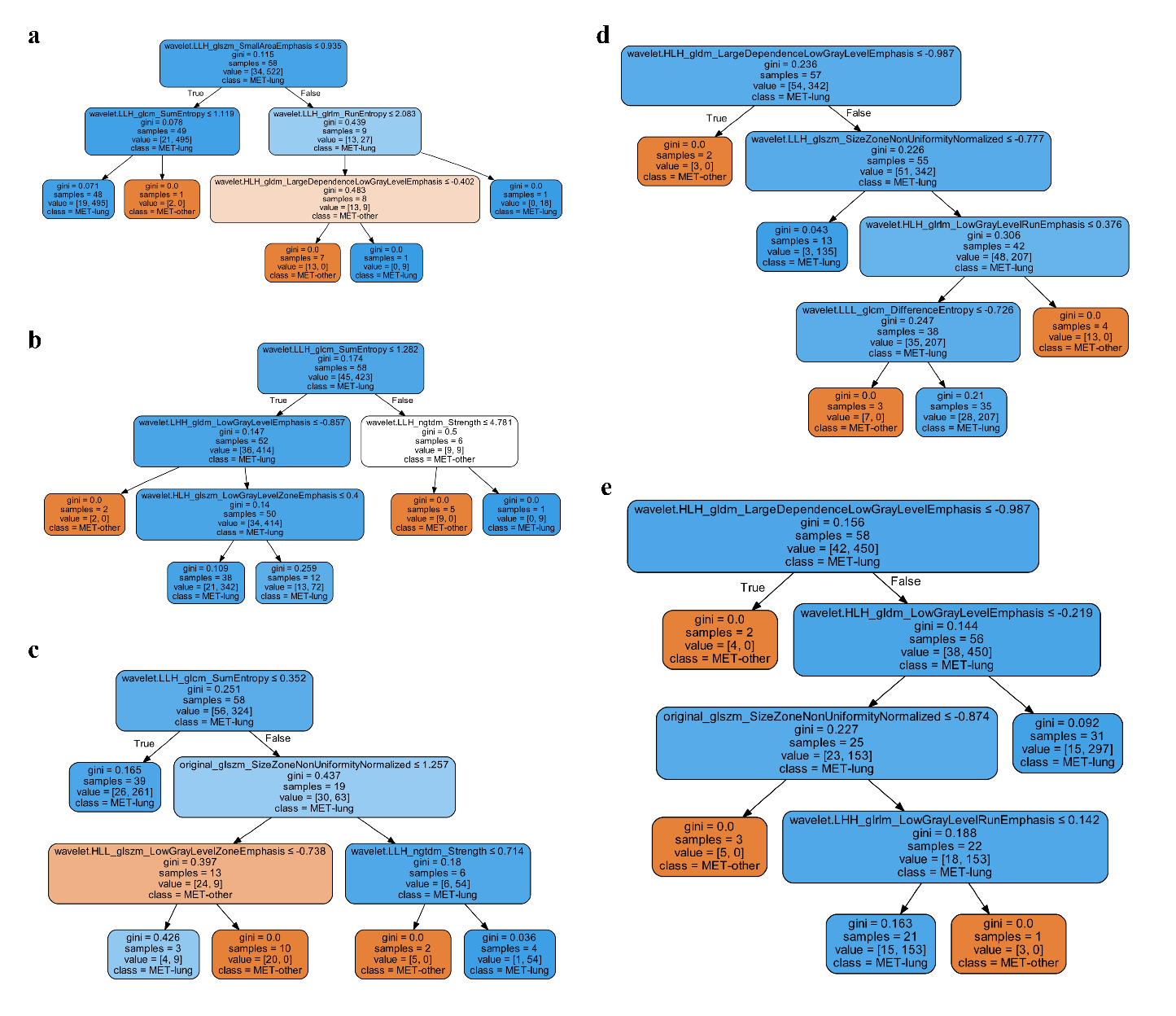


Fig. S5 relief_RF model for differentiation of MET-lung and MET-other.

## S9: Violin figure for each model


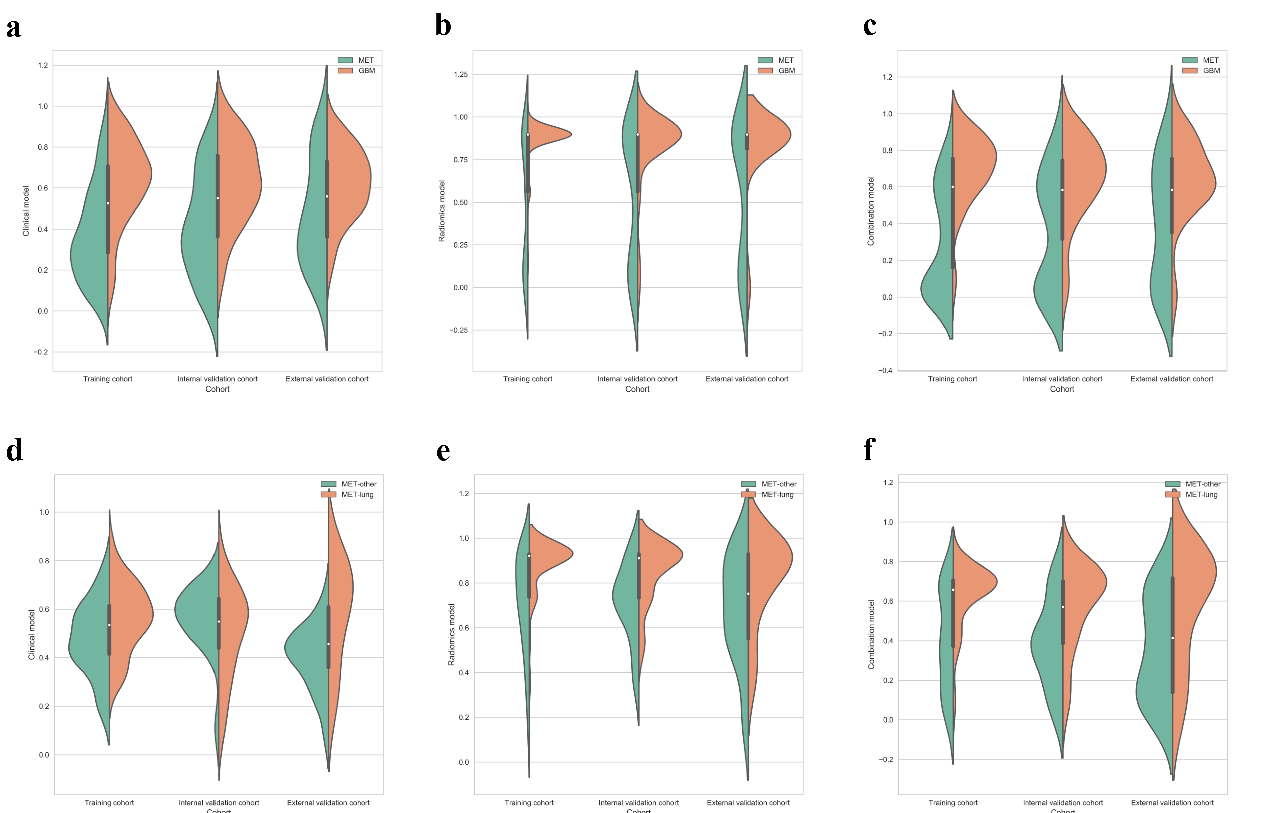


Fig. S6 The violin figure for each model. a-c: models for differentiation of GBM and MET; d-f: models for differentiation of MET-lung and MET-other.

## S10: Correlation between clinical-radiological characteristics and radiomic features


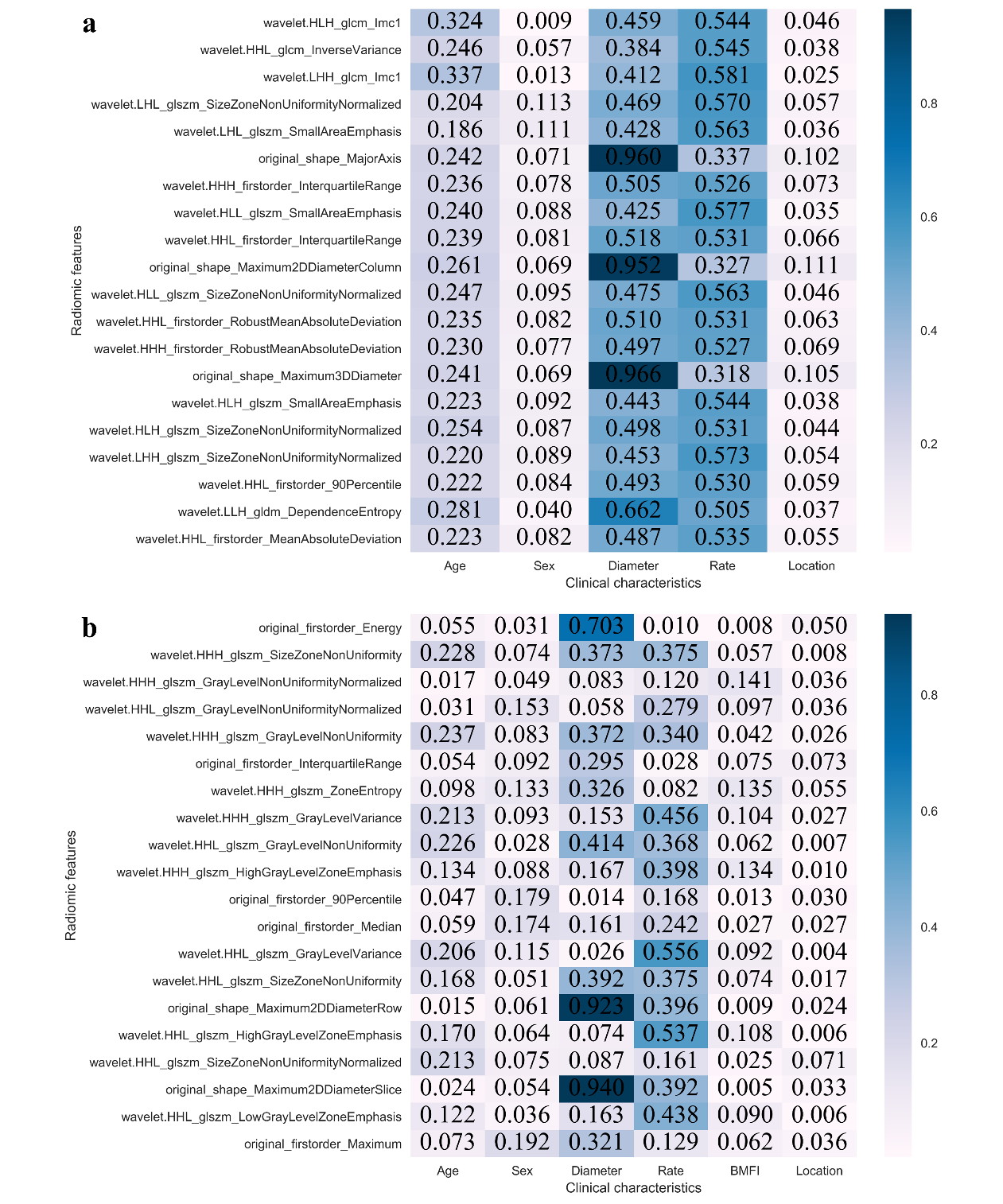


Fig. S7 a) clinical-radiological characteristics and the top 20 features of reliefF method used in distinguishing GBM and MET; b) clinical-radiological characteristics the top 20 features of MCFS method used in distinguishing MET-lung and MET-other.
